# Worsening respiratory failure in an adult hydrocephalic patient with a ventriculo‐pleural shunt

**DOI:** 10.1002/rcr2.660

**Published:** 2020-09-26

**Authors:** Edmond Wong, Vishnu Jeganathan, Samuel Wreghitt, Gavin Davis, Hari Wimaleswaran, Mark E Howard

**Affiliations:** ^1^ Department of Respiratory and Sleep Medicine Austin Health Heidelberg VIC Australia; ^2^ Institute for Breathing and Sleep Heidelberg VIC Australia; ^3^ Department of Neurosurgery Austin Health Heidelberg VIC Australia; ^4^ Faculty of Medicine, Dentistry and Health Sciences University of Melbourne Parkville VIC Australia; ^5^ Turner Institute for Brain and Mental Health Monash University Clayton VIC Australia

**Keywords:** Cerebrospinal fluid shunts, hydrothorax, pleural effusion, respiratory insufficiency

## Abstract

Ventriculo‐pleural (VPL) shunt insertion is performed in hydrocephalic patients when alternative sites of cerebrospinal fluid (CSF) diversion are contraindicated. These include patients with peritoneal complications from ventriculo‐peritoneal shunts. Despite its utility, VPL shunts are uncommon. Hydrothoraces should be considered as a potential cause of dyspnoea in the setting of a VPL shunt. We present a case of worsening respiratory failure in the setting of a massive CSF hydrothorax in a hydrocephalic patient with a VPL shunt to highlight this potential complication of pleural CSF diversion, and present a potential management strategy in patients with premorbid underlying lung pathology. In this case, the hydrothorax was drained and the shunt was converted to ventriculo‐atrial (VA) shunt.

## Graphical abstract

We present a case of an adult hydrocephalic patient with a ventriculo‐pleural shunt in situ presenting with worsening respiratory failure in the setting of a massive cerebrospinal fluid hydrothorax.

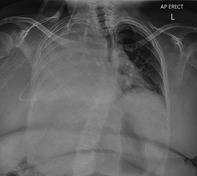

## Introduction

Ventriculo‐peritoneal (VP) shunt insertion is commonly performed in the management of communicating hydrocephalus, mitigating the risk of rising intracranial pressure and cerebral herniation through diversion of cerebrospinal fluid (CSF) to the peritoneal cavity [1]. VP shunts have reported failure rates between 30% and 50% over two years [2, 3]. An alternative to VP shunting includes ventriculo‐pleural (VPL) shunting to allow anterograde flow of CSF into the pleural space. We present a case of an adult hydrocephalic patient with a VPL shunt in situ presenting with worsening respiratory failure in the setting of a massive CSF hydrothorax.

## Case Report

A 44‐year‐old female was referred after a telehealth conference with a six‐month history of worsening dyspnoea, orthopnoea, and increasing non‐invasive ventilation (NIV) dependence (from 10 h per day to 23 h per day). She became profoundly dyspnoeic when NIV was withdrawn for meals and showers. There were no preceding infective symptoms. The patient denied headaches, nausea, vomiting, and had no change in her conscious state.

Her past medical history included cerebral palsy, type 2 diabetes mellitus, and previous pulmonary embolism. She was treated with NIV for chronic ventilatory failure due to central and obstructive sleep apnoea, kyphoscoliosis, and chronic right hemi‐diaphragm elevation secondary to hemidiaphragm paralysis. Spirometry four years prior to presentation in 2016 revealed severe restriction, with a forced expiratory volume in 1 sec (FEV_1_) of 0.43 L (17% predicted), forced vital capacity (FVC) of 0.44 L (15% predicted), and forced expiratory ratio (FER) of 84%.

Her surgical history included a ventriculo‐atrial (VA) shunt inserted at age two years for hydrocephalus of unclear aetiology. Due to growth‐related catheter migration, a revision was made to a VP shunt 12 years later. Following abdominal sepsis secondary to appendiceal perforation, the VP shunt was revised to a VPL shunt at age 26 years.

Previous chest X‐rays (CXR) at age 36 years had identified the presence of a moderate right‐sided pleural effusion. However, given the absence of respiratory symptoms and concern regarding the risk of shunt infection, the fluid was not sampled at that time.

On examination, oxygen saturations were maintained at 100% on room air on NIV (inspiratory positive airway pressure (IPAP): 22 cm H_2_O and expiratory positive airway pressure (EPAP): 7 cm H_2_O). These settings were unchanged prior to and during the course of her illness. She was haemodynamically stable with no features of a tension hydrothorax. Breath sounds were absent over the right lung field. The patient was clinically euvolaemic.

**Figure 1:**
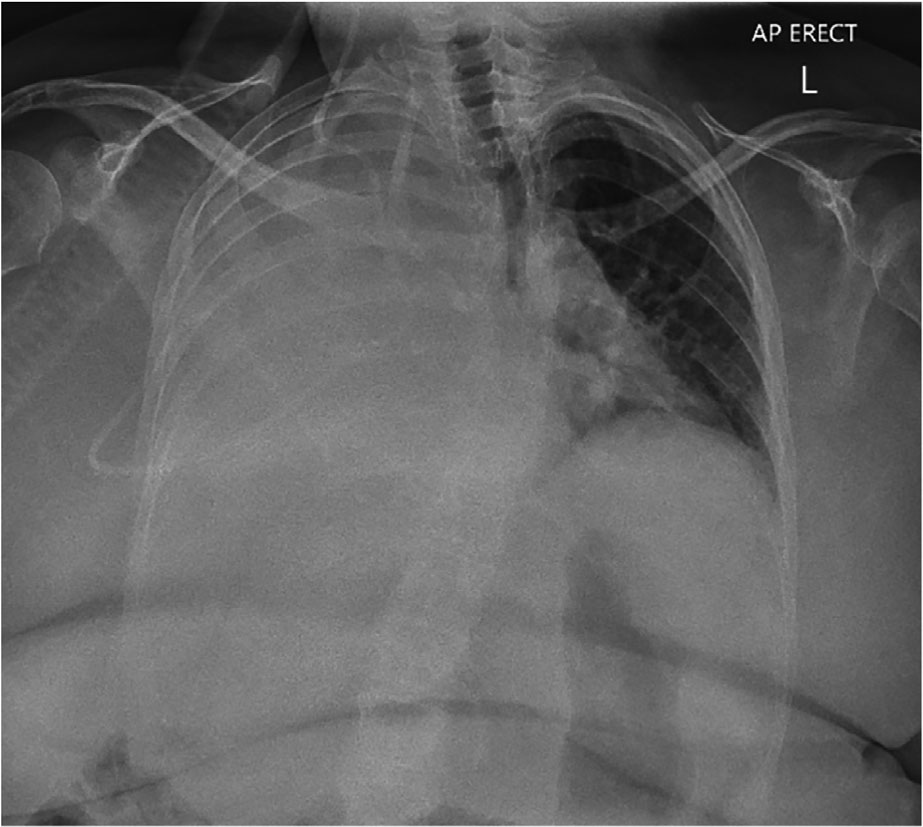
Antero‐posterior chest X‐ray (CXR) showing a large right‐sided pleural effusion due to ventriculo‐pleural (VPL) shunt.

A CXR demonstrated complete opacification of the right hemi‐thorax (Fig. 1). A subsequent computed tomography (CT) scan of her chest revealed a large‐volume, simple appearing pleural effusion with evidence of midline shift. There was no evidence of a blocked VPL shunt clinically or radiologically. Her CT‐brain demonstrated no new enlargement of the cerebral ventricles. Blood analysis revealed white cell count of 10.4 × 10^9^/L (4.0–12.0 × 10^9^/L), C‐reactive protein (CRP) of 15.2 mg/L (<5.0 mg/L), brain natriuretic peptide (BNP) of 69 ng/L (<125 ng/L), albumin of 37 g/L (35–52 g/L), and creatinine of 28 μmol/L (45–90 μmol/L). A transthoracic echocardiogram demonstrated normal systolic function with mildly elevated pulmonary artery pressures of 34 mmHg (<25 mmHg). These results suggested that an infective, cardiac, hepatic, or renal cause for the pleural effusion were unlikely differentials.

The pleural fluid was drained via a 14‐Fr pigtail catheter, with a total output of 2.5 L of light yellow, serous fluid. Biochemical results displayed a total protein of <30 g/L and lactate dehydrogenase (LDH) of 90 U/L, consistent with a transudative effusion by Light et al.'s criteria [4]. Pleural fluid β‐2 transferrin was positive, indicating the presence of CSF. The pleural fluid was culture negative, and cytology did not identify the presence of malignant cells.

A CT‐chest performed following removal of the drain showed no pulmonary nodules or masses.

Following the drainage, the patient's dyspnoea improved and she no longer required daytime NIV. Her respiratory rate was 18 breaths per minute, and her oxygen saturations were 100% on room air. Subsequent arterial blood gas analysis showed a pH of 7.40 (7.35–7.45) and partial pressure of carbon dioxide (PaCO_2_) was within normal limits at 36 (35–45 mmHg).

Given the risk of contamination, the VPL shunt was then externalized, with a daily CSF output of 250 mL. Following a multidisciplinary discussion between the neurosurgical team, respiratory unit, intensive care unit and anaesthetics, the VPL shunt was then surgically converted to a VA shunt by connecting a new distal catheter to the shunt system, and introducing this to the right atrium via the right subclavian vein. The post‐operative course was uncomplicated.

## Discussion

Hydrocephalus occurs more commonly in the paediatric population, with reports that those younger than 18 years constitute 77% of all hydrocephalic patients worldwide [5]. In patients who proceed to CSF diversion surgery, the survival rates are 69% and 60% at 10 and 20 years, respectively [6]. With improved medical provision and early recognition of complications, an increasing proportion of paediatric patients with hydrocephalus are transitioning into adulthood. Thus, the recognition of a hydrothorax in an adult patient with previous VPL shunting reporting shortness of breath is important.

VPL shunt insertion as an alternative to VP shunting was first reported by Ransohoff in 1954 [7]. Since then, over a hundred cases of VPL shunt insertion and at least 50 pleural effusions as a complication have been reported [8]. The true incidence of VPL shunt utilization today is unknown, and therefore the ensuing rates of pleural effusions as a complication are unreported.

CSF hydrothoraces have been infrequently described in the literature. Their heterogeneous presentations include gradual worsening of dyspnoea to acute respiratory failure from a tension hydrothorax [9, 10]. Symptom onset has been documented to be between five days and 19 years post shunt insertion [11, 12]. In our patient, increasing ventilatory failure and ventilator dependence occurred 18 years after VPL shunt insertion. The largest shunt series from Megison and Benzel described 88 VPL shunt procedures in adults with four (4.5%) developing pleural effusions, and an overall complication rate of 24% which also included subdural haematoma, subdural effusions, pneumothorax, catheter migration, and infection [13]. A smaller single‐centre case series reported on 19 adult patients who received VPL shunting—at five‐year follow‐up, three pleural effusions were diagnosed, of which two were symptomatic and required surgical revision. This suggests an 11% incidence of pleural effusions in adult hydrocephalic patients [14]. This differs from Hoffman et al. who demonstrated a pleural effusion prevalence of 20% in 59 children undergoing VPL shunt insertion [15]. Hence, pleural effusions as a complication of VPL shunting are common in both the adult and paediatric populations and can occur many years after shunt insertion. Given that many of these studies are old, it is unclear whether advances in modern medicine have led to changes in the incidence of pleural CSF accumulation.

Pleural fluid analysis of CSF hydrothoraces is consistent with a transudative effusion. There is characteristically low protein, low LDH, low glucose, and positive β‐2 transferrin in biochemical analysis [16]. Furthermore, the macroscopic appearance is water‐like [17].

Thoracocentesis in patients with CSF hydrothoraces should be considered in the presence of symptoms, respiratory failure, or large‐volume effusions. Exudative effusions such as empyema and malignancy require shunt relocation to mitigate the risk of local transmission of pathogens or malignant cells from pleura to CSF. In this case, the VPL shunt was externalized given the potential infection risk whilst performing thoracocentesis. The decision to subsequently convert the externalized shunt to a VA shunt following respiratory recovery was influenced by the patient's underlying respiratory disease, ongoing high CSF output in the externalized drain, and complex previous abdominal surgeries.

VA shunt insertion has been performed in adult patients with normal pressure hydrocephalus (NPH), sub‐arachnoid haemorrhage, and malignancy [17]. Hung et al. reported a comparison of VP and VA shunts in adult patients with idiopathic NPH, and found that VA shunts were associated with fewer shunt obstructions and less likely to require shunt revision [18]. Complications of VA shunts can include infection and interstitial nephritis (mechanism thought to be complement‐mediated immune complex deposition) [17, 19]. Kluge et al. reported a 100% survival rate at eight years in 575 patients with VA shunts [20]. The right atrium of the heart thus provides a safe, alternative site for CSF diversion in hydrocephalic patients where other sites are unavailable.

Pleural effusions following VPL shunting is common. Thus, it should be considered as an important differential diagnosis in patients with VPL shunts who present with shortness of breath. The indication for thoracocentesis and shunt relocation are complex decisions that should involve a multidisciplinary team approach between the treating team and neurosurgical unit with operator experience. In adult patients with hydrocephalus and concomitant lung disease requiring VP shunt revision, VA shunt placement may be considered preferential to VPL shunt.

### Disclosure Statement

Appropriate written informed consent was obtained for publication of this case report and accompanying images.
